# Vascular Health of Females with History of Assisted Reproductive Technology

**DOI:** 10.3390/jcdd11020066

**Published:** 2024-02-18

**Authors:** Pengzhu Li, Magdalena Langer, Theresa Vilsmaier, Marie Kramer, Franziska Sciuk, Brenda Kolbinger, André Jakob, Nina Rogenhofer, Robert Dalla-Pozza, Christian Thaler, Nikolaus Alexander Haas, Felix Sebastian Oberhoffer

**Affiliations:** 1 Division of Pediatric Cardiology and Intensive Care, University Hospital, LMU Munich, 81377 Munich, Germany; pengzhu.li.extern@med.uni-muenchen.de (P.L.); nikolaus.haas@med.uni-muenchen.de (N.A.H.); 2 Division of Gynecological Endocrinology and Reproductive Medicine, Department of Gynecology and Obstetrics, University Hospital, LMU Munich, 81377 Munich, Germany

**Keywords:** infertility, assisted reproductive technologies, females, vascular health

## Abstract

The use of assisted reproductive technologies (ART) for the treatment of infertility is gaining popularity. Limited data on the overall vascular health of females with history of ART are available. This pilot study aimed to investigate the overall vascular health of females with history of ART compared to individuals who conceived spontaneously. The assessment of overall vascular health included the measurement of brachial blood pressure, central blood pressure, and pulse wave velocity, as well as the evaluation of the arterial stiffness and carotid intima-media thickness (cIMT) of the common carotid arteries. Conventional blood lipids including lipoprotein a (Lp(a)) were also determined. In total, 45 females with history of ART and 52 females who conceived spontaneously were included (mean age: 47.72 ± 5.96 years vs. 46.84 ± 7.43 years, *p* = 0.525). An initial comparison revealed a significantly higher prevalence of elevated Lp(a) in ART females (*p* = 0.011). However, after multiple comparison correction, the significant result disappeared (*p* = 0.132). Within the cohort of ART females, no significantly higher cardiovascular risk was detected regarding vascular function. The potentially higher prevalence of elevated Lp(a) in ART females must be further investigated in future studies, as it might contribute to the impaired reproductive process in this cohort.

## 1. Introduction

Infertility refers to the inability of a person or a couple to achieve a clinical pregnancy after 12 months or more of regular unprotected sexual intercourse [1]. Infertility remains highly prevalent, with approximately 48 million couples worldwide suffering from it [2]. Several conditions can cause female infertility such as tubal factor infertility, ovulatory dysfunction, and endometriosis; however, in 40% of cases, the underlying cause remains uncertain [3]. People affected by infertility may seek a fertility treatment, from which assisted reproductive technologies (ART) are widely chosen. According to the latest report of the International Committee for Monitoring Assisted Reproductive Technology, more than 3 million ART cycles were performed in 2018 [4]. Different approaches to ART exist, including in vitro fertilization (IVF), intracytoplasmic sperm injection (ICSI), and gamete intrafallopian transfer (GIFT) [5].

In the past, adverse health effects of ART on the offspring’s cardiovascular health were reported [6,7,8]. It was suggested that children born via ART have increased cardiovascular morbidity, but the pathophysiological reasons behind this remain unclear [9]. In addition, it has been proposed that infertile females might display an increased cardiovascular risk [10,11]. Murugappan et al. demonstrated that compared to their fertile peers, infertile females display a higher risk of atherosclerotic cardiovascular disease [10]. Moreover, Farland et al. found that females with a history of infertility have a 1.13-times higher risk of developing cardiovascular diseases compared to controls [11]. Females who suffer from infertility and thus seek ART treatment could potentially pass down certain cardiovascular risk factors to their offspring. However, to the best of our knowledge, limited data on the overall vascular health of females with history of ART are available.

Therefore, we conducted a pilot study aiming to investigate the overall vascular health of females with history of ART compared to individuals who conceived spontaneously.

## 2. Materials and Methods

### 2.1. Ethical Approval

This study was approved by the Ethics Committee of the Medical Faculty of LMU Munich on 27 December 2020 (Ethikkommission der Medizinischen Fakultät der Ludwig-Maximilians-Universität München, Pettenkoferstraße 8a, 80336 Munich, Germany; approval number: 20-0844). The study was conducted following the Declaration of Helsinki. Prior written informed consent was obtained from all study participants.

### 2.2. Study Design

Females who were treated at the Division of Gynecological Endocrinology and Reproductive Medicine, Division of Obstetrics and Gynecology, University Hospital, LMU Munich (Munich, Germany) and successfully conceived a child between 1995 and 2017 with the help of ART were informed in writing about the ongoing study. Age-matched individuals who conceived spontaneously were enrolled by public calls (e.g., flyers and posters in schools, sports clubs, etc.) within the greater Munich area (Germany).

As this pilot study was part of the Munich heARTerY-study (assisted reproductive technologies and their effects on heart and arterial function in youth), inclusion and exclusion criteria focused primarily on the pediatric cohort [12,13,14,15,16]. To assess whether parents with history of ART displayed an increased cardiovascular morbidity, no exclusion criteria were applied for the parental cohort.

Study participants were examined at the Division of Pediatric Cardiology and Intensive Care, University Hospital, LMU Munich (Munich, Germany) from May 2021 to March 2022.

### 2.3. Medical History, Physical Examination, Course of Pregnancy and Birth, Level of Education

The self-reported medical history of each study participant, focusing on the presence of cardiovascular disease (e.g., arterial hypertension, disorders of glucose, and/or lipid metabolism), was assessed. In addition, self-reported smoking status and the regular use of medication were evaluated. All study participants underwent a physical examination. Body weight (kg), height (cm), waist circumference (cm), and hip circumference (cm) were measured in all subjects. The ratio of waist to hip circumference was determined. In addition, body mass index (BMI, kg/m^2^) was calculated. The weight classification was defined as follows: underweight if BMI < 18.5 kg/m^2^, normal weight if BMI ≥ 18.5 kg/m^2^ but <25 kg/m^2^, overweight if BMI ≥ 25 kg/m^2^ but <30 kg/m^2^, and obese if BMI ≥ 30 kg/m^2^ [17]. The following data regarding the course of pregnancy and birth were obtained from electronic and/or maternity records: conception mode (IVF, ICSI, GIFT, spontaneous conception), maternal age at birth (years), BMI at conception (kg/m^2^), presence of multiple pregnancy, weeks of gestation (weeks), maternal blood pressure during pregnancy ≥ 140/90 mmHg. Self-reported maternal educational level was determined according to the German education system: no school leaving qualification (0), lower secondary school leaving certificate (1), intermediate secondary school leaving certificate (2), general qualification for university entrance (3), completed apprenticeship (4), completed university degree (5).

### 2.4. Adherence to the Mediterranean Diet

High adherence to the Mediterranean diet has a positive effect on cardiovascular morbidity [18]. To assess participants’ adherence to the Mediterranean diet, the validated 14-item Mediterranean diet assessment tool developed by Martínez-González et al. was translated into German and applied [19]. According to Martínez-González et al., a score ≤ 7 was considered low adherence to the Mediterranean diet and a score > 7 high adherence to the Mediterranean diet [19].

### 2.5. Level of Physical Activity and Sedentary Behavior

To determine the level of physical activity in study participants, the German version of the Global Physical Activity Questionnaire (GPAQ) officially provided by the World Health Organization (WHO) was used [20]. Picture cards were presented for each activity type [20]. Total and recreational metabolic-equivalent (MET) minutes per week were calculated according to GPAQ recommendations [20]. Adult subjects met WHO recommendations if ≥600 total MET-minutes per week were achieved [20]. Furthermore, study participants were asked how many times per week muscle-strengthening activities were performed and how much time was spent per day with sedentary activities.

### 2.6. Vascular Function

#### 2.6.1. Pulse Wave Analysis

An oscillometric blood pressure device (Mobil-O-Graph^®^, IEM GmbH, Aachen, Germany) was utilized to measure brachial systolic blood pressure (SBP, mmHg), brachial diastolic blood pressure (DBP, mmHg), mean arterial pressure (MAP, mmHg), heart rate (HR, bpm), central SBP (cSBP, mmHg), central DBP (cDBP, mmHg), augmentation index averaged to a heart rate of 75 bpm (AIx@75, %), and pulse wave velocity (PWV, m/s). Elevated SBP and DBP were present if SBP ≥ 130 mmHg and DBP ≥ 85 mmHg [21]. Cuff sizes were chosen based on participants’ right upper arm circumference. Study participants were asked to remain in a supine and calm position ≥ 5 min before and during the examination. Three consecutive measurements were performed and averaged.

#### 2.6.2. Sonography of the Common Carotid Artery

Sonography of both common carotid arteries (CCA) was performed by one investigator for all study participants using either a Philips iE33 xMatrix or a Philips Epiq 7G ultrasound device (Philips Healthcare, Amsterdam, The Netherlands). During the examination, study participants were asked to remain in a supine position, and the neck was extended to a 45° angle and turned to the opposite side of examination [22]. Offline analysis was conducted by one investigator.

##### Peak Circumferential Strain, Peak Strain Rate and Arterial Distensibility

The area directly below the carotid bifurcation was examined in the short-axis view using a 3–8 MHz sector array transducer (Philips Healthcare, Amsterdam, The Netherlands). Three consecutive loops were recorded under three-lead ECG tracing and transferred to a separate workstation (QLAB Cardiovascular Ultrasound Quantification Software, version 11.1, Philips Healthcare, Amsterdam, The Netherlands) for offline analysis. The software’s SAX-A function was utilized. The vascular region of interest (ROI) was manually adjusted to precisely track the vessel’s wall and to avoid tracking of the perivascular tissue. Pixels of the vascular ROI were then tracked two-dimensionally over the cardiac cycle. Peak circumferential strain (CS, %) and peak strain rate (SR, 1/s) were determined manually. To improve data validity, the average of three measurements was computed for each CCA side. Arterial distensibility (mmHg^−1^ × 10^−3^) was calculated using the following formula [23]:

$$\mathrm{Arterial}\;\mathrm{Distensibility}=\frac{2\times \mathrm{Peak}\;\mathrm{Circumferential}\;\mathrm{Strain}}{\mathrm{Systolic}\;\mathrm{Blood}\;\mathrm{Pressure}-\mathrm{Diastolic}\;\mathrm{Blood}\;\mathrm{Pressure}}$$

In addition, CS, SR, and arterial distensibility of the right and left CCA were averaged.

##### Carotid Intima-Media Thickness

At the level of carotid bifurcation, both CCAs were evaluated in long-axis view utilizing a 3–12 MHz linear array transducer (Philips Healthcare, Amsterdam, The Netherlands). Three consecutive loops were recorded under three-lead ECG tracing and transferred to a separate workstation (QLAB Cardiovascular Ultrasound Quantification Software, version 11.1, Philips Healthcare, Amsterdam, The Netherlands) for offline analysis. At end-diastole (R wave in ECG), the carotid intima-media thickness (cIMT, mm) was assessed semi-automatically for each side. The ROI was set proximal to the carotid bifurcation and the length was adjusted to 10 mm. Three measurements were performed on each side, and the average cIMT for the right and left CCA was calculated. Moreover, the mean cIMT value of both CCAs was assessed.

##### Stiffness Index β

The abovementioned sonographic study protocol was applied. M-mode examinations of both CCAs were performed in long-axis view under three-lead ECG tracking using a 3–12 MHz linear array transducer (Philips Healthcare, Amsterdam, The Netherlands). The end-diastolic diameter (dD, mm) and end-systolic diameter (sD, mm) of both CCAs were measured offline on a separate workstation (IntelliSpace Cardiovascular Ultrasound Viewer, Philips Healthcare, Amsterdam, The Netherlands).

Stiffness index β was defined as [23]:

$$\mathrm{Stiffness}\;\mathrm{Index}\;\beta =\frac{\ln\left(\frac{\mathrm{SBP}}{\mathrm{DBP}}\right)}{\Delta D/\mathrm{dD}}$$

### 2.7. Blood Lipid Profile

To evaluate the blood lipid profile, total cholesterol (TC, mg/dL), low-density lipoprotein cholesterol (LDL-C, mg/dL), high-density lipoprotein cholesterol (HDL-C, mg/dL), non-high-density lipoprotein cholesterol (non-HDL, mg/dL), triglycerides (mg/dL), and lipoprotein a (Lp(a), mg/dL) were assessed. A fasting period of ≥4 h was requested before blood drawing. The presence of elevated conventional blood lipids was defined according to adult recommendations [24,25]. A Lp(a) ≥ 50 mg/dL was defined as increased [26].

### 2.8. Statistical Analysis

As this was a pilot study, a prior sample size calculation was not feasible. A chi-square test was applied to compare nominal data. Continuous parameters were tested for normality using the Kolmogorov–Smirnov test and the Shapiro–Wilk test. In case of normal distribution, an unpaired t-test was used. For non-normally distributed continuous variables, the Mann–Whitney U test was utilized. Normally distributed data are presented as means ± standard deviation (SD) and non-normally distributed data as medians (interquartile range (IQR)). To control the false discovery rate (FDR), the Benjamini–Hochberg (BH) procedure was applied for multiple comparison correction. For data analysis, SPSS 26 (IBM SPSS Statistics for Windows, version 26.0, IBM Corp., Armonk, NY, USA) was used. A *p* < 0.05 was considered statistically significant.

## 3. Results

### 3.1. Patient Characteristics

A total of 46 ART females and 52 controls were initially recruited for this study. One subject in the ART group was excluded due to insufficient data assessment. In total, 45 ART females and 52 individuals who conceived spontaneously were included in the final analysis.

Mean age was 47.72 ± 5.96 years in ART females and 46.84 ± 7.43 years in controls (*p* = 0.525). The two groups did not differ significantly in anthropometric variables, weight classification, smoking status, or educational level (Table 1).

A total of 34 ART females conceived successfully with the help of ICSI, 10 with the help of IVF, and 1 with the help of GIFT. Regarding the course of pregnancy and birth, maternal age at birth and the presence of multiple pregnancy were significantly higher in the ART cohort compared to controls (Table 1).

Self-reported medical history was not significantly different between ART females and their peers (Table 1).

### 3.2. Diet Quality, Level of Physical Activity, and Sedentary Behavior

The two groups did not differ significantly in diet quality, level of physical activity, or sedentary behavior (Table 2).

### 3.3. Vascular Function

Vascular function did not show a significant difference between ART females and controls (Table 3).

### 3.4. Blood Lipid Profile

An initial comparison revealed a significantly higher prevalence of elevated Lp(a) in ART females (*p* = 0.011). However, after multiple comparison correction, this significant result no longer remained (*p* = 0.132) (Table 4). The remaining blood lipid profile did not display significant alterations between the two groups (Table 4).

## 4. Discussion

This pilot study investigated overall vascular health in 45 ART females and 52 controls who conceived spontaneously. Interestingly, the results of this study did not reveal significant differences in overall vascular function between ART females and controls. Initially, a significantly higher prevalence of elevated Lp(a) was observed in ART females. However, after multiple comparison correction, this significant result disappeared. Special care was taken to adequately match both groups by age, as well as by diet quality, physical activity, and sedentary behavior. The two groups did not differ significantly in anthropometric variables, weight classification, smoking status, educational level, or self-reported medical history. Maternal age at childbirth was significantly higher in the ART group than in the control group. There is evidence that as age increases, the mother’s cardiovascular system may be less able to adapt to pregnancy, putting the child at higher risk of adverse pregnancy outcomes, which could potentially harm the cardiovascular function of the offspring [27,28]. In addition, the prevalence of multiple pregnancy was significantly higher in the ART group. This may be due to the ART procedure itself, as multiple embryos are often transferred at once. The literature suggests that one in five IVF cycles results in a multiple pregnancy [29]. Even though no significant difference in gestational hypertension was found in our study, multiple pregnancy still resulted in a higher rate of peripartal morbidities, such as preeclampsia [30].

To assess overall vascular function, different non-invasive methodologies were applied, including the measurement of brachial blood pressure, central blood pressure, and PWV via an oscillometric blood pressure device. Additionally, arterial stiffness and cIMT of the CCA were assessed via sonography in this study.

Female infertility can be caused by a variety of factors such as ovulation disorders, hormonal imbalances, structural alterations of the reproductive system, chronic illnesses, lifestyle, and age [2,31]. Some studies have demonstrated that female infertility and cardiovascular dysfunction might have potential associations [32,33]. A recent prospective cohort study of more than 100,000 participants with 28 years of follow-up demonstrated that females with a history of infertility (12 months of trying to conceive without success, including individuals who subsequently conceived) may show an increased risk of facing coronary heart disease later in life compared to fertile controls [11]. The study observed that cardiovascular risk correlated inversely with age of first infertility diagnosis. Interestingly, the authors revealed that cardiovascular risk may be restricted to infertility-related ovulation disorders such as polycystic ovary syndrome (PCOS) and endometriosis [11]. Another cross-sectional study including over 700 female participants from the USA (age range: 20–59 years) demonstrated that experiencing infertility at any point within the female reproductive window may be linked with later-life cardiovascular morbidity [34].

In contrast to the abovementioned studies, we did not find a significant difference in vascular function between individuals who conceived through ART and control subjects who conceived spontaneously. One of the reasons for this might be that our study solely focused on females who had successfully conceived offspring with the help of ART, while other studies focused on females with infertility who did not conceive successfully even when an ART history was potentially present [11,32]. Therefore, we may have examined individuals with “less-pronounced” infertility. Additionally, the subjects in our cohort displayed a younger age compared to the study participants of Farland et al. [11]; hence, vascular abnormalities might have not become detectable yet in our study.

Initially, a significantly higher prevalence of elevated Lp(a) was observed in ART females. However, after multiple comparison correction, this significant result disappeared. Lp(a) has a similar structure to low-density lipoprotein (LDL) and is characterized by the binding of apolipoprotein(a) (apo (a)) to apolipoprotein B100 [35]. Circulating Lp(a) levels are largely determined by the *LPA* gene encoding apo(a) and are therefore not significantly affected by age, sex, physical activity, and diet [36]. Elevated Lp(a) levels are present in 10% to 30% of the population and increase cardiovascular risk [37,38,39]. Limited data on the relationship between Lp(a) and infertility are available. However, some studies suggest a link between elevated Lp(a) levels and female disorders that are associated with infertility, such as endometriosis and PCOS. Crook et al. investigated the blood lipid profile of 29 females with endometriosis and 29 healthy females and found that the Lp(a) levels in females with endometriosis were significantly higher [40]. Furthermore, Swetha et al. demonstrated that the prevalence of elevated Lp(a) levels was significantly higher in PCOS patients [41].

The exact pathophysiological mechanisms linking elevated Lp(a) levels and female infertility are still largely unknown and require further investigation. Potentially, elevated Lp(a) levels disturb the regulation of female sex hormones, such as estrogen and progesterone, and lead to irregular menstrual cycles, anovulation, and ultimately decreased fertility [42,43]. Moreover, it was shown that elevated Lp(a) levels induce inflammatory responses via interleukin 6 (IL-6), which could potentially alter cardiovascular, as well as reproductive, function [44].

With the widespread use of ART, it is of particular concern whether the offspring of females with history of ART may inherit certain cardiovascular risk factors associated with female infertility, such as altered Lp(a) levels. Interestingly, some studies have suggested that ART offspring may have a higher risk of vascular dysfunction [6,45]. Therefore, further studies investigating the role of maternal risk factors in cardiovascular morbidity of ART offspring are required.

Notably, the causes of female infertility can be complex and are often multifactorial. Although there are shared risk factors between the onset of cardiovascular disease and infertility (e.g., obesity, arterial hypertension, diabetes) [46,47,48,49], research on the association between female infertility and increased cardiovascular risk is sparse. Hence, in the future, more studies are required addressing such pathophysiological interactions.

### 4.1. Limitations

#### 4.1.1. Study Design and Study Population

The present study was a single-center study in Germany. The participants were recruited in the greater Munich area, and therefore the results might be restricted by cultural factors, socioeconomic status (SES), and ethnic homogeneity, due to selection bias. Leischik et al. pointed out that the spectrum of diseases is different in countries with different levels of development. In developed countries, non-communicable diseases, especially cardiovascular diseases, have received much attention, and differences in SES can lead to health inequalities. People with higher SES tend to have better health outcomes than those with lower SES [50]. Moreover, different ecosystems and the physical environment affect health performance, including the cardiovascular system [50]. The sample size of this pilot study could be regarded as relatively small. Our ART group included different types of ART, including IVF, ICSI, and GIFT, which resulted in a relatively heterogeneous study sample. This manuscript does not address data on the number of ART cycles in the examined females or the type of embryo transfer (fresh vs. frozen) conducted. Some patient characteristics were obtained via questioning the participants. Hence, some response bias might be present. Females with history of ART were more frequently on hormone replacement therapy, which could have altered the results on vascular function [51]. We did not acquire data on an individual’s menstrual cycle, which could potentially alter cardiovascular function. Furthermore, we recruited relatively young ART females, and therefore some cardiovascular changes might have not become detectable yet. The causes of infertility are complex. Hormonal, genetic, and psychological factors, as well as the potential influence of male infertility, were not addressed in this study. One in four females with infertility suffer from hormonal imbalances and disorders causing anovulation [52]. In approximately 10% of cases, genetic abnormalities are present [53]. Moreover, psychological distress may be also related to the development of female infertility [54]. Hence, prospective multicenter studies are needed to validate the results demonstrated in this study and enable precise cardiovascular risk stratification of females with history of ART.

#### 4.1.2. Methodology

As this was a pilot study, a prior power analysis was not feasible. We applied the BH procedure to reduce FDR and this might have lowered the ability to find significant results and reduce statistical effects. This pilot study suggests Lp(a) as a significant cardiovascular risk factor in ART females. To draw more definitive conclusions, a separate study focusing solely on Lp(a) should be executed in the future. In this study, we applied a wide variety of methodologies to detect vascular dysfunction. Although carotid-femoral PWV (cfPWV) measurement is considered the noninvasive gold standard of arterial stiffness assessment, it has not been widely used in clinical practice, because the procedure is time-consuming and requires special equipment. We therefore applied oscillometric PWV measurement, as it has a satisfying agreement and consistency with the abovementioned gold standard, making it an easy as well as suitable screening tool for cardiovascular risk determination [55,56]. To the best of our knowledge, two-dimensional speckle tracking (2DST) is a new technique for measuring arterial stiffness and has not yet been validated. However, several studies have suggested that 2DST may be a useful tool for the noninvasive assessment of arterial stiffness compared to the current gold standard of measuring cfPWV [57,58]. The assessment of cIMT and 2DST parameters was not conducted blindly, which could have resulted in some bias. However, various vascular parameters (e.g., brachial blood pressure, PWV) were measured automatically by the respective devices. To reduce intermeasurement variability in this study, the mean of three measurements was calculated to improve data validity. To reduce interobserver variability, ultrasound images were acquired and analyzed offline by a single investigator.

## 5. Conclusions

This pilot study investigated the overall vascular health of females with history of ART compared to individuals who conceived spontaneously. No significant differences in vascular function were displayed between the two groups. The potentially higher prevalence of elevated Lp(a), a cardiovascular risk factor, among females with history of ART might contribute to an impaired reproductive process and play an important role in the etiology of female infertility. As female infertility has been linked with an increased cardiovascular risk within the literature, subjects suffering from infertility might profit from a cardiovascular screening to identify risk factors at an early stage and therefore improve their overall outcome. In the future, multicenter studies with a larger sample size, as well as a long-term follow-up design, are needed for a more precise cardiovascular risk stratification of infertile females.

## Figures and Tables

**Table 1:** Patients’ characteristics.

Variable	ART (*n* = 45)	Control (*n* = 52)	*p*-Value	*p*-Value Adjusted
Age (years)	47.72 ± 5.96	46.84 ± 7.43	0.525	0.735
Bodyweight (kg)	64.50 (60.90–77.93)	65.15 (59.30–74.18)	0.332	0.719
Height (cm)	167.86 ± 7.14	168.25 ± 6.56	0.782	0.876
BMI (kg/m^2^)	23.41 (21.43–28.90)	23.03 (21.60–25.27)	0.506	0.735
Underweight (*n* (%))	1 (2.22)	1 (1.92)	0.724	0.845
Normal weight (*n* (%))	27 (60.00)	37 (71.16)		
Overweight (*n* (%))	8 (17.78)	7 (13.46)		
Obese (*n* (%))	9 (20.00)	7 (13.46)		
Waist-hip ratio	0.85 (0.82–0.91)	0.84 (0.80–0.88)	0.175	0.663
Smoking (*n* (%))	4 (8.89)	5 (9.62)	1	1
Maternal educational level	4.00 (3.00–5.00)	5.00 4.00–5.00)	0.188	0.663
Course of pregnancy and birth				
Maternal age at birth (years)	38.45 ± 3.64	31.85 ± 3.99	<0.001 ***	0.028 *
BMI at conception (kg/m^2^) ^1^	22.22 (20.23–24.25)	21.42 (20.19–22.82)	0.277	0.705
Multiple pregnancy (*n* (%))	11 (24.44)	2 (3.85)	0.003 **	0.042 *
Weeks of gestation (weeks) ^2^	39.00 (36.00–40.00)	39.00 (38.00–40.00)	0.101	0.663
Maternal blood pressure during pregnancy ≥ 140/90 mmHg ^3^	0 (0)	3 (12.5)	0.246	0.689
Medical history				
Arterial hypertension (*n* (%))	6 (13.33)	4 (7.69)	0.506	0.735
Dyslipidemia (*n* (%))	4 (8.89)	7 (13.46)	0.479	0.735
Glucose metabolism disorder (*n* (%))	2 (4.44)	0 (0)	0.213	0.663
Thyroid disease (*n* (%))	9 (20)	10 (19.23)	0.924	0.995
History of thrombosis (*n* (%))	2 (4.44)	1 (1.92)	0.595	0.756
History of pulmonary embolism (*n* (%))	1 (2.22)	0 (0)	0.464	0.735
History of questionable transient ischemic attack (*n* (%))	1 (2.22)	0 (0)	0.464	0.735
History of cancer (*n* (%))	0 (0)	2 (3.85)	0.497	0.735
Medication				
Antihypertensive medication (*n* (%))	1 (2.22)	3 (5.77)	0.621	0.756
Lipid-lowering medication (*n* (%))	1 (2.22)	3 (5.77)	0.621	0.756
L-thyroxine (*n* (%))	9 (20)	10 (19.23)	0.924	0.958
Antidiabetic medication (*n* (%))	2 (4.44)	0 (0)	0.213	0.663
Blood thinners (*n* (%))	2 (4.44)	0 (0)	0.213	0.663
Hormone replacement therapy (*n* (%))	5 (11.11)	0 (0)	0.019 *	0.177
Oral contraceptives (*n* (%))	3 (6.67)	1 (1.92)	0.334	0.719

ART, assisted reproductive technologies; BMI, body mass index. ^1^ 33 ART females and 36 control subjects were included in the analysis. ^2^ 41 ART females and 48 control subjects were included in the analysis. ^3^ 21 ART females and 24 control subjects were included in the analysis. Maternal educational level was assessed according to the German education system: no school leaving qualification (0), lower secondary school leaving certificate (1), intermediate secondary school leaving certificate (2), general qualification for university entrance (3), completed apprenticeship (4), completed university degree (5). Data are presented as means ± SD for normally distributed parameters and as medians (IQR) for non-normally distributed parameters. Nominal data are presented as *n* (%). Benjamini–Hochberg procedure was applied for adjusting *p*-value. * *p* < 0.05, ** *p* ≤ 0.01, *** *p* ≤ 0.001.

**Table 2:** Diet quality, level of physical activity, and sedentary behavior.

Variable	ART (*n* = 45)	Control (*n* = 52)	*p*-Value
MEDAS	6.49 ± 2.29	6.87 ± 2.28	0.420
Total MET-min per week ^1^	2880.00 (1350.00–8490.00)	3300.00 (925.00–5670.00)	0.843
Recreational MET-min per week ^1^	900.00 (240.00–2070.00)	1080.00 (375.00–1680.00)	0.935
Muscle strengthening activities (times/week)	0 (0–1)	0 (0–0)	0.534
Sedentary behavior (hours/day)	6.01 ± 3.53	6.70 ± 2.73	0.281

ART, assisted reproductive technologies; MEDAS, Mediterranean diet adherence score; MET, metabolic-equivalent. Data are presented as means ± SD for normally distributed parameters and as medians (IQR) for non-normally distributed parameters. Nominal data are presented as *n* (%). ^1^ 44 ART females were included in the analysis.

**Table 3:** Vascular function.

Variable	ART (*n* = 45)	Control (*n* = 52)	*p*-Value
Pulse wave analysis			
SBP (mmHg)	123.49 ± 15.50	120.94 ± 12.35	0.371
Elevated SBP (*n* (%))	11 (24.44)	13 (25)	0.950
DPB (mmHg)	79.29 ± 10.71	78.01 ± 8.70	0.518
Elevated DBP (*n* (%))	12 (26.67)	12 (23.08)	0.683
MAP (mmHg)	99.51 ± 12.36	97.64 ± 9.87	0.411
cSBP (mmHg)	118.33 ± 14.48	115.05 ± 11.38	0.215
cDBP (mmHg)	80.29 ± 10.87	79.01 ± 8.85	0.526
Heart rate (bpm)	61.42 ± 7.98	60.71 ± 8.76	0.680
AIx@75 (%)	17.85 ± 11.05	20.41 ± 12.60	0.293
PWV (m/s)	6.93 ± 0.96	6.78 ± 0.91	0.424
Sonography of the common carotid artery			
CS (%) ^1^	7.18 ± 2.30	7.30 ± 2.19	0.791
SR (1/s) ^1^	1.58 (1.30–2.08)	1.58 (1.27–2.02)	0.917
Arterial distensibility (mmHg^−1^ × 10^−3^) ^1^	333.31 ± 106.93	347.98 ± 116.06	0.526
Stiffness index β ^2^	5.87 (3.97–8.97)	5.24 (3.59–8.11)	0.438
cIMT (mm)	0.55 (0.50–0.65)	0.55 (0.51–0.60)	0.783

ART, assisted reproductive technologies; SBP, systolic blood pressure; DBP, diastolic blood pressure; MAP, mean arterial pressure; cSBP, central systolic blood pressure; cDBP, central diastolic blood pressure; AIx@75, augmentation index averaged to a heart rate of 75 bpm; PWV, pulse wave velocity; CS, peak circumferential strain; SR, peak strain rate; cIMT, carotid intima-media thickness. ^1^ 44 ART females and 51 control subjects were included in the analysis. ^2^ 50 control subjects were included in the analysis. Data are presented as means ± SD for normally distributed parameters and as medians (IQR) for non-normally distributed parameters. Nominal data are presented as *n* (%).

**Table 4:** Blood lipid profile.

Variable	ART (*n* = 45)	Control (*n* = 52)	*p*-Value	*p*-Value Adjusted
TC (mg/dL)	196.00 (180.00–225.00)	189.00 (175.50–214.00)	0.441	0.722
Increased TC (*n* (%))	20 (44.44)	23 (44.23)	0.983	0.983
LDL-C (mg/dL)	121.16 ± 24.33	115.98 ± 30.76	0.366	0.722
Increased LDL-C (*n* (%))	24 (53.33)	24 (46.15)	0.481	0.722
HDL-C (mg/dL)	69.40 ± 18.47	68.81 ± 17.44	0.871	0.983
Decreased HDL-C (*n* (%))	5 (11.11)	6 (11.54)	0.947	0.983
Non-HDL-C (mg/dL)	124.00 (110.50–156.00)	122.00 (105.75–140.75)	0.399	0.722
Increased Non-HDL-C (*n* (%))	22 (48.89)	21 (40.38)	0.400	0.722
Triglycerides (mg/dL)	81.00 (59.50–99.50)	76.50 (56.00–121.75)	0.876	0.983
Increased Triglycerides (*n* (%))	4 (8.89)	8 (15.38)	0.333	0.722
Lp(a) (mg/dL) ^1^	12.00 (5.00–55.75)	7.00 (5.00–17.00)	0.110	0.66
Increased Lp(a) (*n* (%)) ^1^	10 (27.78)	4 (7.69)	0.011 *	0.132

ART, assisted reproductive technologies; TC, total cholesterol; LDL-C, low-density lipoprotein cholesterol; HDL-C, high-density lipoprotein cholesterol; non-HDL-C, non-HDL cholesterol; Lp(a), lipoprotein (a). Data are presented as means ± SD for normally distributed parameters and as medians (IQR) for non-normally distributed parameters. Nominal data are presented as *n* (%). Benjamini–Hochberg procedure was applied for adjusting *p*-value. ^1^ 36 ART females were included in the analysis. * *p <* 0.05.

## Data Availability

The original contributions presented in the study are included in the article, further inquiries can be directed to the corresponding author.
